# An efficient algorithm for energy harvesting in IIoT based on machine learning and swarm intelligence^☆^

**DOI:** 10.1016/j.heliyon.2023.e17622

**Published:** 2023-06-30

**Authors:** Peizhen Xing, Hui Zhang, Morched Derbali, Shebnam M. Sefat, Amal H. Alharbi, Doaa Sami Khafaga, Nor Samsiah Sani

**Affiliations:** a Henan Vocational College of Water Conservancy and Environment, Zhengzhou, 450008, Henan, China; b College of Information Engineering, Zhengzhou University of Technology, Zhengzhou, 450044, China; c Faculty of Computing and Information Technology (FCIT), King Abdulaziz University (KAU), Jeddah, Saudi Arabia; d Department of Computer Science, Independent University, Bangladesh; e Islamic university Centre for scientific research, The Islamic University, Najaf, Iraq; f Department of Computer Sciences, College of Computer and Information Sciences, Princess Nourah Bint Abdulrahman University, P.O. Box 84428, Riyadh, 11671, Saudi Arabia; g Center for Artificial Intelligence Technology, Faculty of Information Science and Technology, Universiti Kebangsaan Malaysia, 43600, Bangi, Selangor, Malaysia

**Keywords:** IoT, Machine learning, Artificial intelligence, Resource optimization

## Abstract

The Internet of Things (IoT) is a network of smart gadgets that are connected through the Internet, including computers, cameras, smart sensors, and mobile phones. Recent developments in the industrial IoT (IIoT) have enabled a wide range of applications, from small businesses to smart cities, which have become indispensable to many facets of human existence. In a system with a few devices, the short lifespan of conventional batteries, which raises maintenance costs, necessitates more replacements and has a negative environmental impact, does not present a problem. However, in networks with millions or even billions of devices, it poses a serious problem. The rapid expansion of the IoT paradigm is threatened by these battery restrictions, thus academics and businesses are now interested in prolonging the lifespan of IoT devices while retaining optimal performance. Resource management is an important aspect of IIoT because it's scarce and limited. Therefore, this paper proposed an efficient algorithm based on federated learning. Firstly, the optimization problem is decomposed into various sub-problems. Then, the particle swarm optimization algorithm is deployed to solve the energy budget. Finally, a communication resource is optimized by an iterative matching algorithm. Simulation results show that the proposed algorithm has better performance as compared with existing algorithms.

## 1 Introduction

With the widespread deployment of 5G technology around the world, industry and academia have begun to explore 6G networks [1]. The 6G network will integrate communication and computing functions, adopt advanced artificial intelligence (AI) technology, and provide intelligent connection services for typical application scenarios such as autonomous driving, virtual reality, smart factories, and smart cities [2]. Traditional resource management mechanisms are difficult to meet the complex requirements of 6G networks. Therefore, the next-generation network based on intelligent endogenous and simplified networks has become a hotspot of extensive research. With the popularization of the Internet of Things (IoT), various services put forward more and higher requirements for communication technology, which cannot be supported by the current 5G network, and the 6G network is expected to promote the industrial revolution and support the new requirements of the IoT, among which wireless distributed computing is 6G enabling technology [3]. As a distributed machine learning method, federated learning can meet the privacy protection and low latency requirements of 6G networks [4], and is a key technology for building 6G IoT systems [5]. Due to the data security and fast data analysis and decision-making requirements of the Industrial Internet of Things (IIoT), federated learning is expected to be applied to the IIoT scenario to train machine learning models [6]. However, there are problems of low resource availability [7] in IIoT scenarios, such as low computing power, limited bandwidth, and battery life, and the heterogeneous properties of device capabilities [8] also increase the performance gap between devices. However, most of the existing research on federated learning for IIoT does not consider issues such as limited battery energy, resulting in limited applicable scenarios [[9], [10]].

Most of the research on federated learning is based on charging equipment [11], which does not need to consider the service life of the equipment. When performing federated learning based on battery-powered devices [12], the device may be offline during training due to battery energy depletion, reducing system performance [13]. Therefore, long-term energy scheduling and energy-saving technologies for batteries are the keys to efficient federated learning for battery-powered IIoT. Existing research on federated learning based on battery-powered devices mainly uses battery life as a device selection indicator [7] or adjusts some training parameters to improve battery life [12], 14–15, such as training batch size, CPU frequency, etc. There is little overall scheduling and allocation of battery energy, and there is a lack of control over battery life from a long-term perspective. Research on energy scheduling applies to limited scenarios. For example, reference [16] allocates energy according to battery power and training rounds, where the training rounds are a fixed value, ignoring the total running time limit in the actual scene, and when the training time is a fixed value It is difficult to determine the training rounds [6], 17 before training, and it is difficult to effectively extend it to the IIoT scenario with a given time budget. Therefore, the federated learning of IIoT based on battery-powered equipment lacks holistic and systematic energy scheduling and energy-saving technology research.

Multiple optimization directions of federated learning, such as time, cost, and accuracy, are difficult to balance [18], so the optimization goal needs to be weighed [[19], [20], [21], [22], [23], [24], [25], [26]]. Existing works have studied the problem of achieving maximum learning accuracy with a fixed training time [[22], [23], [24], [25]], but most of them have not considered the impact of battery power supply and wireless transmission on system performance. Since the complexity of the long-term optimization solution increases exponentially with time [26], an independent iterative optimization method [27] can be used to simplify the problem. At the same time, to balance training time and accuracy goals and speed up learning, learning efficiency [28,29] can be introduced as an optimization goal for resource management. Reference [28] maximizes learning efficiency by joint batch selection and communication resource allocation. Reference [29] proposes a communication resource allocation and data selection algorithm that maximizes learning efficiency.

Aiming at the resource optimization management problems such as heterogeneous device battery energy and communication resource allocation in the low resource availability environment faced by the current research, this paper proposes a resource management algorithm for the federated learning network in the battery-powered IIoT. This paper considers the impact of battery power supply and wireless transmission on federated learning performance and jointly optimizes the management of device resources, communication resources, and battery energy to solve the optimization problem of battery-powered IIoT devices with a fixed training time to achieve maximum learning accuracy.

The main contributions of this paper are as follows.

• A federated learning resource management algorithm for battery-powered IIoT devices is designed. The algorithm transforms long-term optimization into dynamic round-by-round optimization, considering the limited number of resource blocks, wireless channel interference, and limited battery energy, and proposes a mathematical model aimed at learning efficiency optimization. The original optimization issue is separated into three interconnected sub-problems, which are tackled separately such as device, communication, and battery energy resource allocation.
• To solve the learning efficiency optimization problem, the particle swarm optimization algorithm is used to solve the optimal equipment transmission and computing resource allocation strategy under the energy budget, and the expression of the change of the single-round global loss function is deduced, based on which the learning efficiency and communication resource allocation are obtained. The ideal communication resource allocation method is then solved using a resource block iterative matching technique.
• A technique for allocating battery energy is suggested. The training rounds are estimated under the condition of given equipment resources and communication resource allocation, the equipment is screened according to the delay, and the CPU frequency of the equipment is adjusted for energy allocation so that the equipment is not affected by training, it is interrupted when the battery runs out.
• The simulation results show that the proposed algorithm in this paper can obtain better learning performance using battery-powered devices within the training time, and it reflects the effectiveness of the algorithm in this paper compared with the benchmark algorithm.

## 2 System model

**Fig. 1:**
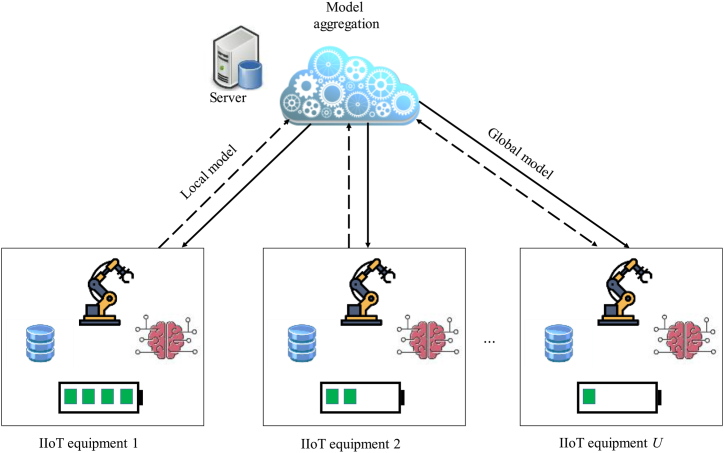
Proposed system model.

The federated learning system model under the IIoT environment considered in this paper is shown in Fig. 1, which contains U devices and a server. Uplink and downlink communication is performed between the device and the server through wireless channels.

### 2.1 Wireless communication model

Since all local models are transmitted through the wireless network, the influence of wireless factors on the performance of federated learning needs to be considered when deploying the federated learning model on the wireless network. Next, the wireless model is constructed from the aspects of transmission delay, energy loss, and transmission success probability.

#### 2.1.1 Transmission delay

Under the considered federated learning system, uplink transmission adopts Orthogonal Frequency Division Multiple Access (OFDMA) technologies, as it still has important reference value in the future 6G wireless network [[30], [31]]. Assuming that M resource blocks in the uplink form a resource block set Ω, the *t*-round training of device *i* transmits its local federated learning model parameters to the base station through resource block *n*, and its uplink rate is expressed in Eq. [1] [[16], [17]]:

(1) $$c_{i,n,t}^{Up}=B^{Up}\;\log\left(1+\frac{P_{i,t}h_{i}}{I_{n}+B^{Up}N_{0}}\right)$$

where $B^{Up}$ is the bandwidth of a single resource block in the uplink; $P_{i,t}$ is the transmission power of device i in round t; $I_{n}$ is the interference of the signal on resource block *n*; $N_{0}$ is the noise power spectral density; $h_{i}$ is the channel gain from device *i* to server.

According to the resource block matching scheme, the training uplink rate of device *i* in round *t* is expressed in Eq. [2]:

(2) $$c_{i,t}^{Up}=\sum_{n=1}^{M}r_{i,n,t}c_{i,n,t}^{Up}$$

Among them, $r_{i,n,t}\in \left\{0,1\right\}$ in $r_{i,n,t}=1$ use resource block *n* for parameter transmission in round *t* training of device *i*. $r_{i,n,t}=0$ on the contrary, do not use resource block *n* for parameter transmission. Assuming that the size of the model parameters is D, the uplink transmission delay of the *t*-round training of the corresponding device *i* through the resource block *n* is expressed in Eq. [3]:

(3) $$T_{i,n,t}^{Up}=\frac{D}{c_{i,n,t}^{Up}}$$

Similarly, the transmission delay of the *t*-round training of device *i* is expressed in Eq. [4]:

(4) $$T_{i,t}^{Up}=\sum_{n=1}^{M}r_{i,n,t}T_{i,n,t}^{Up}$$

Since the device allocates resource blocks for transmission in the uplink, the bandwidth resource is tight, while the downlink uses broadcast transmission, and the bandwidth resource pressure is small, so the fluctuation of the downlink delay is negligible compared with the uplink delay. Define $c^{Down}$ as the downlink rate, and the corresponding downlink delay is expressed Eq. [5]:

(5) $$T^{Down}=\frac{D}{c^{Down}}$$

The device trains the local model locally using its computing resources. The calculation delay of the *t*-round training of device *i* is expressed in Eq. [6] [[17], [18], [19]]:

(6) $$T_{i,t}^{Comp}=\frac{\varepsilon_{i}K_{i}{\bar{\omega }}_{i}}{f_{i,t}}$$

where, $\varepsilon_{i}$, $K_{i}$, and ${\bar{\omega }}_{i}$ are the sizes of the sample data of device *i*, the number of samples of the device, and the number of CPU cycles required to process each bit of data respectively, and $f_{i,t}$ is the CPU frequency of the *t*-th round of training for device *i*.

Combining the three delays, the delay of the *t*-th round of training for device *i* is expressed in Eq. [7]:

(7) $$T_{i,t}=T_{i,t}^{Up}+T_{i,t}^{Comp}+T^{Down}$$

The training delay of each round of federated learning depends on the maximum delay of the selected device, so the delay of the *t*-th round of training is expressed in Eq. [8]:

(8) $$T_{t}=\max_{i\in \Lambda_{t}}\left(T_{i,t}^{Up}+T_{i,t}^{Comp}\right)+T^{Down}$$

Among them, $\Lambda_{t}$ is the equipment set selected in the *t*-th round of training, that is, $\sum_{n=1}^{M}r_{i,n,t}=1,i\in \Lambda_{t}$.

#### 2.1.2 Energy loss

It is assumed that the aggregation server has a sufficient power supply, so the energy consumption on the server is not considered, only the energy consumption of the equipment is concerned. Device energy consumption includes two parts: device local training and wireless transmission energy consumption, so the energy consumption of device *i* is defined as [32]:

(9) $$e_{i,t}=\delta_{i}{\bar{\omega }}_{i}f_{i,t}^{2}\varepsilon_{i}K_{i}+P_{i,t}T_{i,t}^{Up}$$

where, $\delta_{i}{\bar{\omega }}_{i}f_{i,t}^{2}\varepsilon_{i}K_{i}$ is the energy consumption of device *i* in the *t*-th round of training the local model, which is related to the energy consumption coefficient $\delta_{i}$ of the processor of device *i*, the number of CPU cycles ${\bar{\omega }}_{i}$ required per bit of data, the CPU frequency $f_{i,t}$; the size $\varepsilon_{i}$ of the equipment sample data is related to the sample size $K_{i}$; $P_{i,t}T_{i,t}^{Up}$ is the energy consumption of uplink wireless transmission, which is related to the transmission power $P_{i,t}$ and the transmission delay $T_{i,t}^{Up}$.

#### 2.1.3 Transmission success probability

Transmission errors can occur due to interference and noise in the wireless channel. The expression of the average packet error rate of the data packet transmission system on the quasi-static fading channel is given in [33], the waterfall threshold *m* is given, and the packet error rate of the resource block *n* in the *t*-round training of the device *i* is defined in Eq. [10]:

(10) $$Error_{i,n,t}=1-e^{\left(\frac{-m\left(I_{n}+B^{Up}N_{0}\right)}{P_{i,t}h_{t}}\right)}$$

where, $P_{i,t}h_{i}/I_{n}+B^{Up}N_{0}$ is the signal-to-interference-noise ratio (SNR).

Assuming that a local model in the uplink is transmitted using a packet, and the transmission failure will not be retransmitted the success probability of device *i* transmitting the local model under resource block *n* in round *t* is expressed in Eq. [11]:

(11) $$q_{i,n,t}=e^{\left(\frac{-m\left(I_{n}+B^{Up}N_{0}\right)}{P_{i,t}h_{i}}\right)}$$

At the same time, the success probability of device *i* training and transmission local model in round *t* s, $q_{i,t}=\sum_{n=1}^{R}r_{i,n,t}q_{i,n,t}$. The binary variable $s_{i,t}$ that defines whether the parameter upload is successful or not according to the probability of successful transmission is expressed in Eq. [12]:

(12) $$s_{i,t}=\left\{ \begin{array}{c} 1,p=q_{i,t}^{Up}\\ 0,p=1-q_{i,t}^{Up} \end{array}\right.$$

where, $s_{i,t}=1$ means the upload is successful; $s_{i,t}=0$ means the upload failed.

### 2.2 Federated learning model

Define the device set u={1,2,…,U}, the data collected locally by device *i* is $D_{i}=\left\{d_{i1},d_{i2},\ldots ,d_{iK_{i}}\right\},i\in u$. The number of samples of device i is $K_{i}$, and the sum of the number of training samples of the device is expressed in Eq. [13]:

(13) $$K=\sum_{i=1}^{U}K_{i}$$

Assume that the output of data $d_{ik}$ is $o_{ik}$ and the output vector of device i is $O_{i}=\left\{o_{i1},o_{i2},\ldots ,o_{ik}\right\}$. After the local model is sent to the server, it will be integrated into a global model $g_{t}$ and sent back to the IIoT device. Therefore, the updated expression of the global model $g_{t}$ considering device selection and transmission failure is expressed in Eq. [14]:

(14) $$g_{t}=\frac{\sum_{i=1}^{U}K_{i}a_{i,t}s_{i,t}w_{i,t}}{\sum_{i=1}^{U}K_{i}a_{i,t}s_{i,t}}$$

where, $a_{i,t}=\sum_{n=1}^{R}r_{i,n,t}$ represents whether the device is selected.

Define the loss function of each data as $f\left(g_{t},d_{ik},o_{ik}\right)$, and the local loss function of device *i* is defined as the mean value of the local data loss is expressed in Eq. [15]:

(15) $$F_{i}\left(g_{t}\right)=\frac{\sum_{k=1}^{K_{t}}f\left(g_{t},d_{ik},o_{ik}\right)}{K_{i}}$$

The overall loss function of all devices is the mean value of the local losses of all devices, defined in Eq. [16]:

(16) $$F\left(g_{t}\right)=\frac{\sum_{i=1}^{U}F_{i}\left(g_{t}\right)}{K}$$

For the sake of simplicity, the loss function $f\left(g_{t},d_{ik},o_{ik}\right)$ of the *k*-th data of the *t*-th round of training of device *i* is abbreviated as $F_{i,k}\left(g_{t}\right)$ in the following text.

After the device receives the global model $g_{t}$, the local model update formula is expressed in Eq. [17]:

(17) $$w_{i,t+1}=g_{t}-\lambda \nabla F_{i}\left(g_{t}\right)$$

where λ is the learning rate. Further, the updated formula of the global model loss function is:

(18) $$g_{t+1}=g_{t}-\lambda \left(\nabla F\left(g_{t}\right)-v_{t}\right)$$

Among them, $v_{t}$ is the difference between the ideal global gradient and the actual global gradient regardless of device selection and transmission failure, defined in Eq. [19]:

(19) $$v_{t}=\nabla F\left(g_{t}\right)-\nabla F^{\prime }\left(g_{t}\right)$$

Among them, the actual global model gradient $\nabla F^{\prime }\left(g_{t}\right)$ is expressed in Eq. [20]:

(20) $$\nabla F^{\prime }\left(g_{t}\right)=\frac{\sum_{i=1}^{U}K_{i}a_{i,t}s_{i,t}\nabla F_{i}\left(g_{t}\right)}{\sum_{i=1}^{U}K_{i}a_{i,t}s_{i,t}}$$

### 2.3 Problem model

Aiming at the optimization problem of obtaining the maximum learning accuracy for a device with limited power within a fixed training time τ, since the maximum learning accuracy can be transformed into the minimum loss value of the global model, the optimization objective can be expressed in Eq. [21]:

(21) $$P1:\min\;F\left(g_{w}\right)$$

where, the device battery energy limit condition is, $\sum_{t=0}^{W}e_{i,t}\leq E_{i},\forall i\in u$; $e_{i,t}$ is the energy consumption of device *i* defined in the Eq. [9] in the *t*-th round of training; $E_{i}$ is the total energy of the battery, W For the maximum training rounds within the training time τ, the total energy consumption of the device should be less than the total energy of the battery.

P1 represents the minimization of the global loss function in training time under a given energy budget. Since the device is powered by batteries, there is a long-term resource budget constraint, and if the device runs out of energy, it will not be able to participate in subsequent training. To solve P1, it is necessary to know the total training rounds W in the training time, but it is difficult to determine before training [6, 17], and P1 is a nonlinear programming problem, and the complexity of solving is exponential with the increase of rounds W growth [26]. In addition, the delay $T_{i,t}$, and sending state $s_{i,t}$ vary with the iteration round index t, and due to the iterative nature of federated learning, the global model is related to the training of all past rounds [24]. In summary, it is difficult to optimize device resources for a long time. Therefore, this paper divides the long-term power budget into the available power budget for each round, and takes the learning efficiency [28–29] as the optimization goal of each round, aiming to accelerate federated learning training and quickly minimize the global loss function.

Learning efficiency is defined as the ratio of global loss decay to time delay per round, which represents the global loss decay rate, which is the learning rate of the model. Among them, the *t*-th round of global loss decay is defined as the difference between the (t−1)-th round of training global model $g_{t-1}$ loss value $F\left(g_{t-1}\right)$ and the *t*-th round loss value $F\left(g_{t}\right)$, which can be expressed in Eq. [22]:

(22) $$\Delta F_{t}=F\left(g_{t-1}\right)-F\left(g_{t}\right)$$

Therefore, according to the definition of learning efficiency, the learning efficiency $A_{t}$ of the *t*-th round of training can be expressed as:

(23) $$A_{t}=\frac{\Delta F_{t}}{T_{t}}$$

where $T_{t}$ is the training delay of the *t*-th round.

In this paper, independent iterative optimization is carried out round by round, and the problem of maximizing learning efficiency $A_{t}$ in round t is dynamically solved. Combined with training constraints, the optimization problem P1 can be transformed into:

(24) $$P2:\max_{R_{t},P_{t},f_{t},E_{t}}A_{t}$$

(24a) $$s.t.\;a_{i,t},r_{i,n,t}\in \left(0,1\right),\forall i\in u,n\in \Omega$$

(24b) $$\sum_{n=1}^{M}r_{i,n,t}=a_{i,t}\text{,}\forall i\in u$$

(24c) $$\sum_{i=1}^{U}r_{i,n,t}=1\text{,}\forall n\in \Omega$$

(24d) $$0\leq P_{i,t}\leq P_{i,\max},\forall i\in u$$

(24e) $$f_{i,\min}\leq f_{i,t}\leq f_{i,\max},\forall i\in u$$

(24f) $$e_{i,t}\leq E_{i,t},\forall i\in u$$

where, the total energy consumption of the device satisfies, $\sum_{t=0}^{W}e_{i,t}\leq E_{i},\forall i\in u$, that is, the sum of the energy consumption of the device *i* under the total training rounds W is less than the total energy $E_{i}$ of the battery. Equation [24] represents the optimized communication resource allocation matrix for the *t*-th round $R_{t}=\left[r_{1,t},\ldots ,r_{U,t}\right],r_{i,t}=\left[r_{i,1,t},\ldots ,r_{r,M,t}\right]\text{;}$ device transmission power vector $P_{t}=\left[P_{1,t},\ldots ,P_{U,t}\right]$; CPU frequency vector $f_{t}=\left[f_{1,t},\ldots ,f_{U,t}\right]$, and energy budget allocation vector $E_{t}=\left[E_{1,t},\ldots ,E_{U,t}\right]$ to maximize the learning efficiency $A_{t}$. $P_{i,\max}$, $f_{i,\max}$, and $f_{i,\min}$ are the maximum transmit power, maximum and minimum CPU frequencies of device *i* respectively. Formulas (24a), (24b), (24c), (24d) constrain each device to only occupy one resource block for uplink data transmission. Formula [24d] constraints the maximum transmit power. Formula [24e] constraints the CPU frequency, and formula [24f] constraints the maximum Training energy consumption, $E_{i,t}$ is the maximum energy consumption budget of device *i* in round t.

To quantify the impact of wireless transmission on federated learning performance, this paper introduces the following lemma to describe the mathematical relationship between global loss attenuation, transmission success rate, and device scheduling.

Lemma 1 The lower bound on the expected global loss decay $\Delta F_{t}$ in training t is given by Eq. [25]:

(25) $$E\left(\Delta F\right)\geq \frac{1}{2L}{\left\|\nabla F\left(g_{t}\right)\right\|}^{2}\left(1-\frac{4UB^{2}}{K^{2}}\sum_{i=1}^{U}K_{i}^{2}\left(1-a_{i,t}\left(2q_{i,t}-q_{i,t}^{2}\right)\right)\right)$$

where, L is the smoothing parameter, set as the reciprocal of the learning rate, that is, $L=\frac{1}{\lambda }$ [[34], [35], [36]]; B is a strongly convex parameter; E(⋅) is the expectation about the launch success rate.

Proof: See Appendix 1

## 3 Proposed algorithm

Considering the complexity of solving multi-dimensional joint variables, this paper decouples problem P2 into equipment resource allocation sub-problem, communication resource allocation sub-problem, and battery energy allocation sub-problem, and solves it by variable iteration [18–19, 23]. First, solve the optimal CPU frequency and transmit power under the known energy budget, and obtain the resource allocation strategy. Secondly, the communication resource allocation strategy is solved under the known equipment resource allocation strategy. Thirdly, the next round of device battery energy allocation strategy is estimated according to the communication resource allocation strategy. Finally, multiple iterative optimizations are performed until the end of the training time τ.

### 3.1 Device resource allocation

To solve optimization problem P2, it is necessary to balance the transmission energy and calculate the energy allocation of the equipment under the equipment energy consumption budget of each round. In this paper, the transmission energy is related to the transmission power, and the calculation energy is related to the CPU frequency. Given the communication resource allocation matrix $R_{t}$ and the energy budget allocation vector $E_{t}$, optimize the device transmission power vector $P_{t}$ and the CPU frequency vector $f_{t}$ to maximize the learning efficiency $A_{t}$, the above problem is an NP-hard problem, and it is difficult to obtain the optimal solution. Therefore, an approximate solution algorithm is designed to approach the global optimal allocation strategy.

First, based on the mathematical relationship between transmission success rate and global loss attenuation in Lemma 1, it can be known that the influence of transmission success rate on global loss attenuation is non-linear, that is, the greater the transmission success rate, the smoother the change of global loss attenuation. Secondly, based on the definition formula [11] of the transmission success rate, it can be seen that since the transmission success rate has nothing to do with the CPU frequency, the relationship between the transmission success rate and the transmission success probability is the e-exponential distribution of the negative reciprocal. Under ideal channel conditions, the transmission success probability does not change significantly within the general value range of the transmission power. Therefore, under general conditions, the change in the global loss attenuation caused by the change of the transmit power is not obvious, and the CPU frequency has nothing to do with the global loss attenuation. At the same time, according to the delay definition formula [7], it can be seen that the device delay is related to both the CPU frequency and the transmission power, and the calculation delay is positively related to the reciprocal of the CPU frequency. Based on Equation [23], it can be seen that learning efficiency is defined as the ratio of global loss attenuation to time delay.

Based on the above reasons, the time delay $T_{t}$ in learning efficiency is more sensitive to the change of CPU frequency and transmission power than the global loss attenuation $\Delta F_{t}$. To reduce the complexity of the solution, the problem of maximizing the learning efficiency is transformed into the problem of minimizing the time delay, to obtain the approximate solution of the transmit power vector $P^{*}$ and the approximate solution of the CPU frequency vector $f^{*}$. At the same time, after the original device resource allocation sub-problem is approximated as the delay minimization problem since there is no coupling relationship between devices, the problem can be further decoupled and the difficulty of solving can be reduced, and multi-threaded parallel computing can be used to reduce the solution of the device resource allocation sub-problem time. Specifically, the equipment resource allocation problem P2.1 is:

$$P2.1:\min_{f_{i,t,}P_{i,t}}\left(\frac{D}{B^{Up}\;\log\left(1+\frac{P_{i,t}h_{t}}{I_{n}+B^{Up}N_{0}}\right)}+T^{Down}+\frac{\varepsilon_{i}K_{i}{\bar{\omega }}_{i}}{f_{i,t}}\right)$$

(26) s.t. Eq. (24d) ∼ (24f)

To solve problem P2.1, this paper degenerates the solution of the original problem into a simple one-variable function maximization problem by introducing Lemma 2.

Lemma 2 Given the energy budget of the current round, if the energy budget does not support the device to run at the maximum transmit power and CPU frequency, problem P2.1 minimizes the delay of the device transmit power and CPU frequency under the inequality constraint [24f] to take equal sign, that is, $e_{i,t}=E_{i,t}$, so the transmission power $P_{i,t}$ can be expressed by energy budget $E_{i,t}$ and CPU frequency $f_{i,t}$.

Proof: See Appendix 2.

Based on Lemma 2, this paper uses the particle swarm optimization algorithm to calculate the minimum value of the unary function to solve the approximate solution of the device resource allocation strategy for all devices and resource block combinations under the t-round energy budget, that is, the approximate solution of the CPU frequency vector $f_{t}^{*}$ and transmit power vector approximate solution $P_{t}^{*}$. Compared with some other swarm intelligence algorithms, the particle swarm optimization algorithm is better at dealing with continuously changing variables and has good global search capabilities. Its inherent parallelism can facilitate distributed computing and speed up the solution [37]. It can use ready-made smart optimization algorithm libraries (such as scikit-opt) to solve it. The complexity of the particle swarm optimization algorithm is O(pq), where p is the number of iterations and q is the scale of the problem. The time complexity of the device resource allocation algorithm is O(UMpq), where U is the number of devices and M is the number of resource blocks. Since the training delays of different devices under different resource blocks are not coupled, they can be decomposed into multi-threaded parallel computing.

### 3.2 Communication resource allocation

When the energy budget and device resource allocation strategy are known, the communication resource allocation problem P2.2 is:

$$P2.2:\max_{R_{t}}\frac{F\left(g_{t-1}\right)-F\left(g_{t}\right)}{\max_{i\in \Lambda_{t}}\left(T_{i,t}^{Up}+T_{i,t}^{Comp}\right)+T^{Down}}$$

(27) s.t. Eq. (24a) ∼ (24c)

#### 3.2.1 Problem analysis

Substituting the lower limit of expected global loss attenuation in Lemma 1 into Equation [23], the lower limit of learning efficiency $A_{t}$ can be obtained in Eq. [28]:

(28) $$A_{t}=\frac{1}{2L}{\left\|\nabla F\left(g_{t}\right)\right\|}^{2}\left(\frac{1-\frac{4UB^{2}}{K^{2}}\sum_{i=1}^{U}K_{i}^{2}\left(1-a_{i,t}\left(2q_{i,t}-q_{i,t}^{2}\right)\right)}{\max_{i\in \Lambda_{t}}\left(T_{i,t}^{Up}+T_{i,t}^{Comp}\right)+T^{Down}}\right)$$

Since L and ${\left\|\nabla F\left(g_{t}\right)\right\|}^{2}$ are global parameters, they have nothing to do with the communication resource allocation strategy. Divide the left and right sides of formula [28] by ${\left\|\nabla F\left(g_{t}\right)\right\|}^{2}/2L$, we can get:

(29) $$A_{t}^{\prime }\geq \frac{1-\frac{4UB^{2}}{K^{2}}\sum_{i=1}^{U}K_{i}^{2}\left(1-a_{i,t}\left(2q_{i,t}-q_{i,t}^{2}\right)\right)}{\max_{i\in \Lambda_{t}}\left(T_{i,t}^{Up}+T_{i,t}^{Comp}\right)+T^{Down}}$$

where $A_{t}^{\prime }=2LA_{t}/{\left\|\nabla F\left(g_{t}\right)\right\|}^{2}$ is the learning efficiency of deformation. Substituting $a_{i,t}=\sum_{n=1}^{M}r_{i,n,t}$ into formula [29], we get Eq. [30]:

(30) $$A_{t}^{\prime }\geq \frac{1-\frac{4UB^{2}}{K^{2}}\sum_{i=1}^{U}K_{i}^{2}\left(1-\sum_{n=1}^{M}r_{i,n,t}\left(2q_{i,t}-q_{i,t}^{2}\right)\right)}{\max_{i\in \Lambda_{t}}\left({\sum_{n=1}^{M}r_{i,n,t}T}_{i,n,t}^{Up}+T_{i,t}^{Comp}\right)+T^{Down}}$$

Simultaneous formula [30] and problem P2.2, then the problem P2.2 is updated as

$$P3:\max_{R_{t}}\frac{1-\frac{4UB^{2}}{K^{2}}\sum_{i=1}^{U}K_{i}^{2}\left(1-\sum_{n=1}^{M}r_{i,n,t}\left(2q_{i,n,t}-q_{i,n,t}^{2}\right)\right)}{\max_{i\in \Lambda_{t}}\left({\sum_{n=1}^{M}r_{i,n,t}T}_{i,n,t}^{Up}+T_{i,t}^{Comp}\right)+T^{Down}}$$

(31) s.t. Eq. (24a) ∼ (24c)

Due to battery energy constraints, local training for all devices in each round will put huge pressure on battery life, so each round of training cannot obtain the gradient norms of all devices, and the communication resource allocation of the current round of the device can only be combined with the training data of the previous round. So the parameter B in formula [31] is unknown before each round of training.

This paper estimates parameter B based on historical values. First, initialize B=1, that is, there is no difference between device data. The *t*-th round of training can be used to calculate the gradient norm of the global model and the local model $B_{t}$, defining $B_{t}=\max_{i}\left(\frac{\left\|\nabla F_{i}\left(g_{t}\right)\right\|}{\left\|\nabla F\left(g_{t}\right)\right\|}\right)$. In the *t*-th round of training, the parameter B is estimated using the exponential smoothing method is expressed as Eq. [32]:

(32) $$B=\left\{ \begin{array}{c} 1,t=0\\ B_{0},t=1\\ \alpha B_{t-1}+\left(1-\alpha \right)B_{t-2},t>1 \end{array}\right.$$

where α is a tuning parameter that adjusts the weight of recent and past values in the estimate.

#### 3.2.2 Resource block iterative matching algorithm

For the solution of problem P3, this paper proposes an iterative matching algorithm for resource blocks. First, problem P2.1 is solved under the combination of all device resource blocks. According to the obtained device resource allocation strategy, all possible training delays are enumerated using the exhaustive method. Then, the Kuhn-Munkres (KM) [38] algorithm is used to iteratively solve the optimal communication resource allocation strategy under each training delay, that is, the resource block matching vector $R_{t}^{*}$ for each round of equipment, until the number of optional equipment is less than u Based on the number of resource blocks M, the optimal solution that maximizes the learning efficiency under all possible training delays is finally obtained by comparison.

The KM algorithm is a weighted bipartite graph matching algorithm. According to formula [31], set the device and resource block bipartite graph C=(uΩ,ε) and the weight of each edge is expressed as Eq. [33]:

(33) $${Ѱ}_{i,n,t}=\left\{ \begin{array}{c} K_{i}^{2}\left(2q_{i,n,t}-q_{i,n,t}^{2}\right)T_{i,t}^{Up}+T^{Down}+T_{i,t}^{Comp}\leq T_{\max}\\ -\infty ,other \end{array}\right.$$

where $T_{\max}$ is the training delay limit. According to the weight of the bipartite graph, the number of optional devices is expressed as Eq. [34]:

(34) $$u=\sum_{i}^{u}R,\exists {Ѱ}_{i,n,t}>0,n\in \Omega$$

Among them, R(∙) is the indicator function of 0–1.

**Fig. 2:**
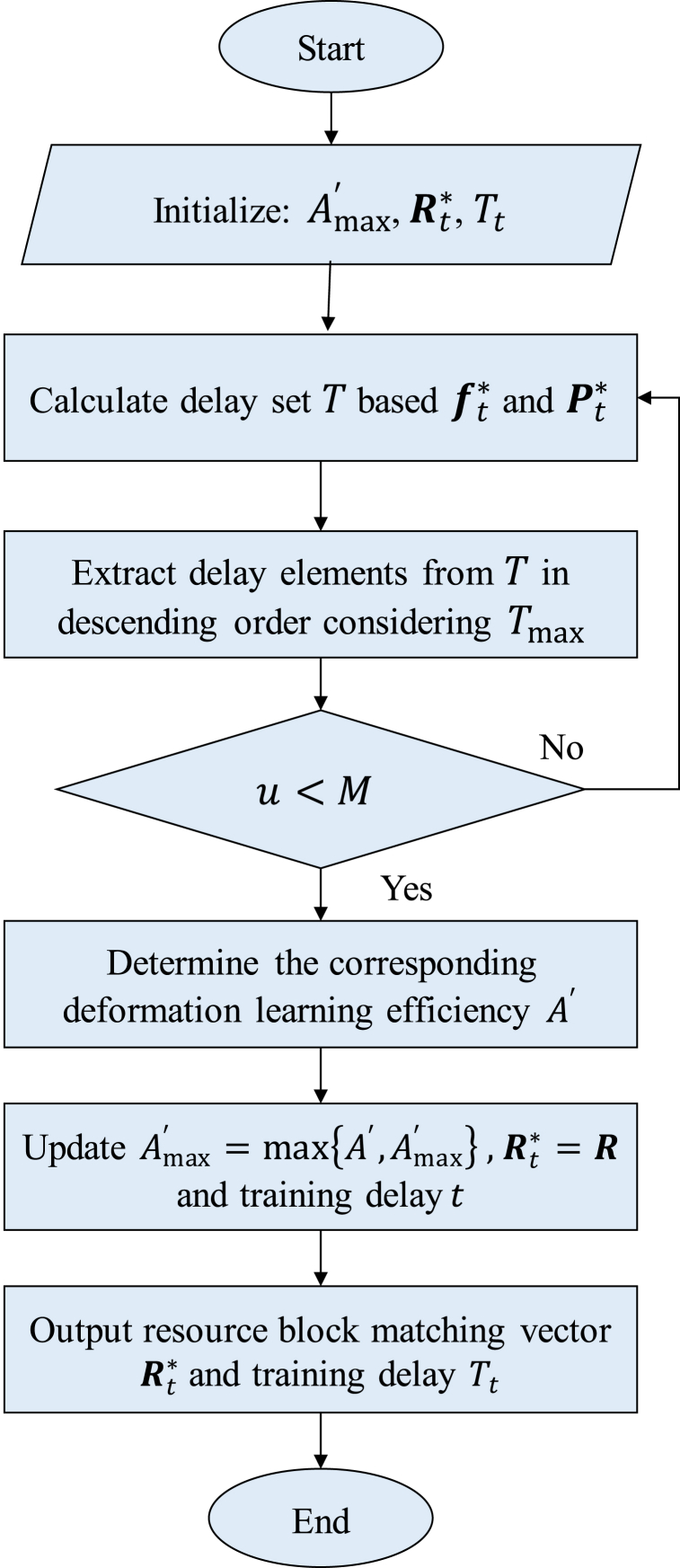
Flowchart of block iterative matching algorithm.

The specific steps of the above process are shown in Fig. 2 and Algorithm 1.

**Table:** 

**Algorithm 1:** Resource Block Iterative Matching Algorithm
**Input:** CPU frequency vector $f^{*}$ and transmit power vector $P^{*}$ Output:*t*-th round resource block matching vector $R_{t}^{*}$ and training delay $T_{t}$ 1: Initialize the upper bound of deformation learning efficiency $A_{\max}^{\prime }=0$, resource block matching vector $R_{t}^{*}$ and training delay $T_{t}$ 2: Calculate the delay set T according to the CPU frequency vector $f_{t}^{*}$ and the transmit power vector $P_{t}^{*}$ 3: Repeat 4: Take out the elements in the set T from large to small as the delay limit $T_{\max}$ 5: If the number u of optional devices under $T_{\max}$ delay is less than the number M of resource blocks 6: If the number of optional devices is insufficient, the loop is terminated 7: The KM algorithm solves the matching vector R of the *t*-th round of candidate resource blocks under the $T_{\max}$ time delay, and calculates the corresponding deformation learning efficiency $A^{\prime }$ 8: Update the upper bound of deformation learning efficiency $A_{\max}^{\prime }=\max\left\{A^{\prime },A_{\max}^{\prime }\right\}$, if $A_{\max}^{\prime }$ is updated to $A^{\prime }$, then update the *t*-th round resource block matching vector $R_{t}^{*}=R$ and training delay t 9: Until all the elements in the delay set T are taken out

The time complexity of the KM algorithm is $O\left(U^{2}M\right)$ [39], so the time complexity of the proposed is $O\left(U^{3}M^{2}\right)$, where U is the number of devices and M is the number of resource blocks. From the expression can be seen that there is no exponential growth relationship between computational complexity and the key variables (i.e., U and M). In addition, communication resource allocation is carried out on the server side, the network computing power is sufficient, and resource block matching schemes under different delay constraints can be decomposed into multi-threaded parallel computing tasks [40].

### 3.3 Battery energy distribution

Due to long-term energy budget constraints, if a device consumes too much energy in a certain round, the device may not be able to participate in aggregation in the future. Therefore, to prolong battery life, this paper allocates and manages battery energy.

At the same time, to make full use of equipment energy, this paper screens the scheduling equipment in each round. If the communication resource allocation is only based on the learning efficiency of each round, it is easy to cause some devices with a small amount of data not to participate in the aggregation for a long time, so the energy of the device cannot be fully utilized. In this paper, it is set that the allocated energy of unaggregated devices can be accumulated and used in each round. When the battery energy of the device is low, the device with the largest delay in the previous round can be removed from the optional equipment in this round. Since the energy of unaggregated devices can be accumulated, subsequent training in the middle, the device will no longer be a delay bottleneck, so in the long run, it will help reduce training delay and improve learning efficiency.

In summary, this paper designs an online battery energy allocation strategy. First, the training rounds are dynamically estimated, combined with the historical accumulated energy, and the energy consumption budget is allocated online for the equipment. Then, a candidate device set is obtained through the last round of device delay, and communication resource allocation is performed according to the candidate device set. Finally, under the given communication resource allocation strategy, the CPU frequency is adjusted to save energy consumption. The strategy is described in detail as follows.

First, the remaining training rounds are estimated based on the remaining training time and the single-round training delay. After the *t*-th round of training, the initial remaining training rounds are estimated in Eq. [35]:

(35) $$W_{t}=\frac{\tau -T_{sum}}{T_{t}}$$

Among them, $T_{t}$ is the *t*-th round of training delay output by Algorithm 1, and $T_{sum}$ is the training time.

To keep the remaining training rounds estimated in different rounds relatively stable, this paper combines historical values to estimate the final remaining training rounds which is expressed as Eq. [36]:

(36) $${\bar{W}}_{t}=\left\{ \begin{array}{c} W_{0},t=0\\ \beta W_{t}+\left(1-\beta \right)W_{t-1},t>0 \end{array}\right.$$

where β is a smoothing constant that characterizes the importance of numerical freshness and is used to adjust the weight of recent and past values in the estimate.

According to the estimated value of the remaining training rounds and the historical accumulated energy, the energy consumption budget of the *t*-round training of equipment *i* can be obtained which is expressed in Eq. [37]:

(37) $$E_{i,t}=\frac{E_{i,t}^{r}}{W_{t-1}}+E_{i,t-1}^{*}$$

Among them, $E_{i,t}^{r}$ is the theoretical remaining total energy of device *i*, which is updated to $E_{i,t}$ after each round of training; $E_{i,t}^{r}=E_{i,t-1}^{r}-E_{i,t}$ is the energy budget of the *t*-th round; $E_{i,t-1}^{*}$ is the unused energy of the (t−1)-th round. The unutilized energy $E_{i,t}^{*}$ of the *t*-round training is the difference between the allocated energy $E_{i,t}$ and the actual energy consumption $e_{i,t}$ in the energy loss model, which can be expressed in Eq. [38]:

(38) $$E_{i,t}^{*}=E_{i,t}-e_{i,t}$$

Secondly, to make full use of device energy, each round of scheduling devices is screened according to the delay to obtain a set of candidate devices for communication resource allocation. In the *t*-th round of training, this paper abandons the selection of the first N devices with the largest delay $T_{i,t-1}$ in the (t−1)-th round, and forms the remaining devices into the *t*-th round candidate device set $\prod_{t}\text{,}$ only allocating communication resource blocks to devices in the set $\prod_{t}\text{.}$ Among them, the number N of abandoned devices is adjusted online according to the feedback of the increase or decrease in learning efficiency.

In the *t*-th round of training, the ${A_{N}^{\prime }}^{mean}$ of the learning efficiency under different numbers of abandoned devices N is expressed in Eq. [39]:

(39) $${A_{N}^{\prime }}^{mean}=\frac{\sum_{t^{\prime }}^{t}l_{N,t^{\prime }}A_{t^{\prime }}^{\prime }}{\sum_{t^{\prime }}^{t}l_{N,t^{\prime }}}$$

Among them, $l_{N,t^{\prime }}$ is a 0–1 variable, $l_{N,t^{\prime }}=1$ means that the number of training abandonment in the *t*′-th round is N, otherwise $l_{N,t^{\prime }}=0$.

To obtain a suitable candidate data set, it is necessary to dynamically adjust the number N of abandoned devices. When the battery energy is insufficient, that is, it does not support the device to train with the maximum transmission power and CPU frequency, and the device screening operation is required. Initially set the number of discarded devices N=1, and after each R round of training, dynamically adjust the number of discarded devices N according to the change in learning efficiency. Given the jump threshold φ of the number of abandoned devices N, when the average learning efficiency increase means ${A_{N}^{\prime }}^{mean}-{A^{\prime }}_{N-1}^{mean}$ or decrease mean ${A^{\prime }}_{N-1}^{mean}-{A^{\prime }}_{N}^{mean}$ exceeds the threshold φ, the number of abandoned devices N corresponds to increase or decrease. After a period of learning, the number N of abandoned devices will tend to be stable or dynamically stable.

Finally, adjust the CPU frequency to save power. Based on Algorithm 1, solve the *t*-round training communication resource block matching vector $R_{t}^{*}$ and the training delay $T_{t}$, to align the time delay of the equipment participating in the aggregation in the *t*-th round of training to the training time $T_{t}$, to eliminate the idle time of the equipment and reduce energy consumption, according to formula [6], the CPU frequency of device *i* is adjusted in Eq. [40]:

(40) $$f_{i,t}=\frac{\varepsilon_{i}K_{i}{\bar{\omega }}_{i}}{T_{t}-T_{i,t}^{Up}-T^{Down}}$$

**Fig. 3:**
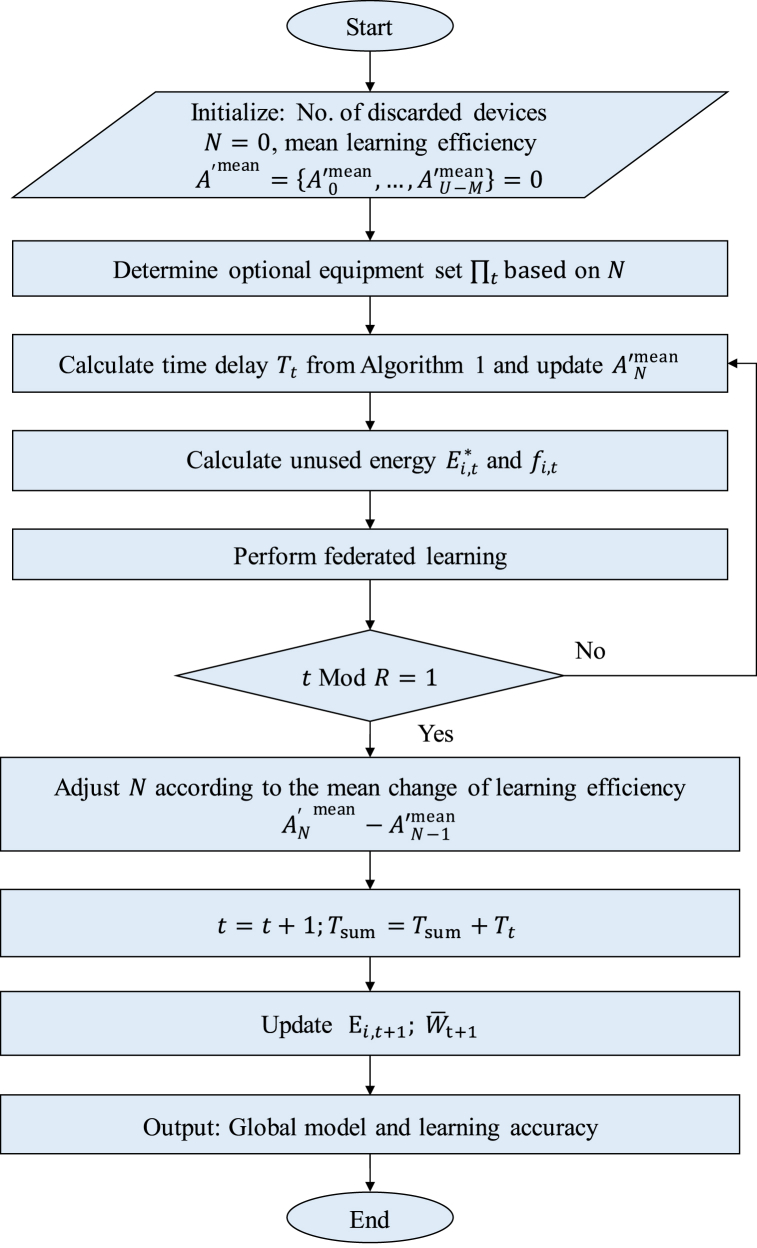
Flowchart of battery energy allocation.

The specific steps of the above process are shown in Fig. 3 and Algorithm 2.

**Table:** 

**Algorithm 2:** Battery energy allocation
**Input:** Equipment total energy consumption budget $E_{i}$ and time budget τ **Output:** Global model and learning accuracy 1: Define t=0, $T_{sum}=0$, initialize the number of discarded devices N=0, and mean learning efficiency ${A^{\prime }}^{mean}=\left\{{A^{\prime }}_{0}^{mean},\ldots ,{A^{\prime }}_{U-M}^{mean}\right\}=0$ 2: Repeat 3: Obtain the optional equipment set $\prod_{t}a$ ccording to the number N of discarded equipment 4: Run Algorithm 1 based on the optional equipment set $\prod_{t}\text{,}$ obtain the time delay $T_{t}$, and update the mean value of learning efficiency ${A^{\prime }}_{N}^{mean}$ 5: Adjust CPU frequency $f_{i,t}$, calculate unused energy $E_{i,t}^{*}$ 6: Perform federated learning training 7: If t Mod R=1 holds 8: Adjust the number of abandoned devices N according to the mean change of learning efficiency ${A_{N}^{\prime }}^{mean}-{A^{\prime }}_{N-1}^{mean}$ 9: Otherwise, continue looping 10: $t=t+1,T_{sum}=T_{sum}+T_{t}$ 11: Update device energy budget $E_{i,t+1}$, and remaining training rounds ${\bar{W}}_{t+1}$ 12: Until the training time sum T exceeds the time budget τ

The battery energy allocation mainly involves energy budget calculation, optional device set acquisition, CPU frequency adjustment, etc. The time complexity of each round of training is O(U).

## 4 Simulation results

To verify the performance of the proposed algorithm, this section simulates and compares different federated learning algorithms in the factory scenario of the battery-powered IIoT.

### 4.1 Simulation scenario and parameters

Taking Fig. 1 as an example, consider a 100 m × 100 m factory environment, where multiple industrial devices are distributed, and there is a server in the center of the factory for model aggregation. The number of equipment U=15, the number of resource blocks M=10, and the interference received by different resource blocks are different. Other simulation parameter settings are shown in Table 1. Using the MNIST data set, the training data is divided into slice data sets with a sample size of 100 numbers in an independent and identical distribution (IID) manner, and different devices are randomly assigned different numbers of slices. Each device uses the divided MNIST dataset to train a simple local three-layer network, which is a hidden layer composed of 512 neurons, a hidden layer composed of 128 neurons, and an output layer. The hidden layer uses ReLU is used as the activation function, a global aggregation is performed after each round of local training, and the loss function adopts the cross entropy function [34].

**Table 1:** Simulation parameters.

Parameter	Value
Processor energy consumption coefficient $\delta_{i}$ of device *i*	$10^{-28}$
Size of a sample data of device *i* ($\varepsilon_{i}/bit$)	24.576
The number of CPU cycles required by device *i* to process each bit of data ${\bar{\omega }}_{i}\left(cycle/bit\right)$	30
Maximum transmit power of device *i*$P_{i,\max}/W$	0.2
Noise power spectral density $N_{0}/\left(dBm/Hz\right)$	−174
Uplink resource block bandwidth $B^{Up}$/MHz	1
Device *i* maximum CPU frequency $f_{i,\max}/GHz$	1–2
Device *i* minimum CPU frequency $f_{i,\min}/GHz$	0.1
Number of IoT nodes	100

To verify the effect of the algorithm proposed in this paper, the algorithm in this paper is simulated and compared with the following benchmark algorithms.

1) Random scheduling algorithm [41]. In each round of communication, M devices are uniformly randomly selected for parameter update, and each selected device is randomly assigned a resource block to transmit the trained parameters. Set the device to use the maximum power and CPU frequency for training, and the device will stop training when the energy is exhausted.
2) Maximum convergence accuracy algorithm [34]. Reference [34] derived the closed-form expression of the expected convergence speed $E\left[F\left(g_{t+1}\right)-F\left(g^{*}\right)\right]$, where $g^{*}$ is based on all optimal federated learning models generated by the local model solves the device selection and resource block matching scheme for the target based on maximizing the convergence accuracy. This scheme requires selecting devices with sufficient power to transmit and update local models throughout federated learning iterations. Set the device to use the maximum power and CPU frequency for training, and the device will stop training when the energy is exhausted.
3) Minimum convergence time algorithm [42]. In [42], the optimization goal is to minimize the training delay. In each round of training, M devices are selected based on the gradient of the local model, and the resource block matching scheme that minimizes the delay is solved for the given device. However, it does not consider the energy limit, and sets the device to use the maximum power and CPU frequency for training, and the device stops training when the energy is exhausted.

In this paper, the total training time is set to 50 s, and the energy consumption budget of the device battery is set to 50 J, 3 J, 2 J, and 1 J in order from large to small, in which 50 J means that the battery is fully charged [6,16,43]. It can support all devices to train at the maximum CPU frequency and maximum sending power all the time.

### 4.2 Result analysis

**Fig. 4:**
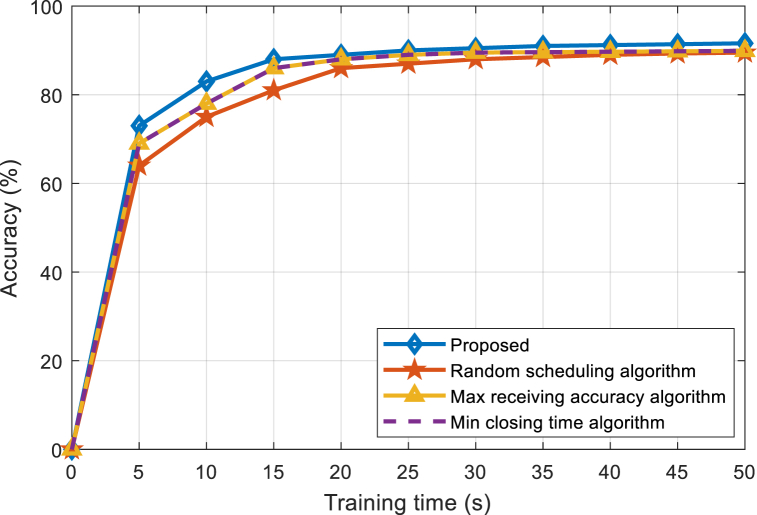
Accuracy comparison between the proposed and existing algorithms under 50 J power.

Fig. 4 shows the performance comparison between the proposed algorithm and the benchmark algorithm under 50 J power. When the battery energy is sufficient, both the proposed algorithm and the benchmark algorithms can run continuously at the maximum CPU frequency and maximum transmission power. It can be seen from Fig. 4 that the proposed algorithm converges faster than the other three benchmark algorithms, and when the training time is long enough, the proposed algorithm is close to the accuracy obtained by the maximum convergence accuracy algorithm. Since the proposed algorithm takes learning efficiency as the optimization goal and aims to improve the training speed when the battery energy is sufficient, the proposed algorithm can converge to the highest accuracy at the fastest speed, and the advantage is more obvious in scenarios where the training time is small.

**Fig. 5:**
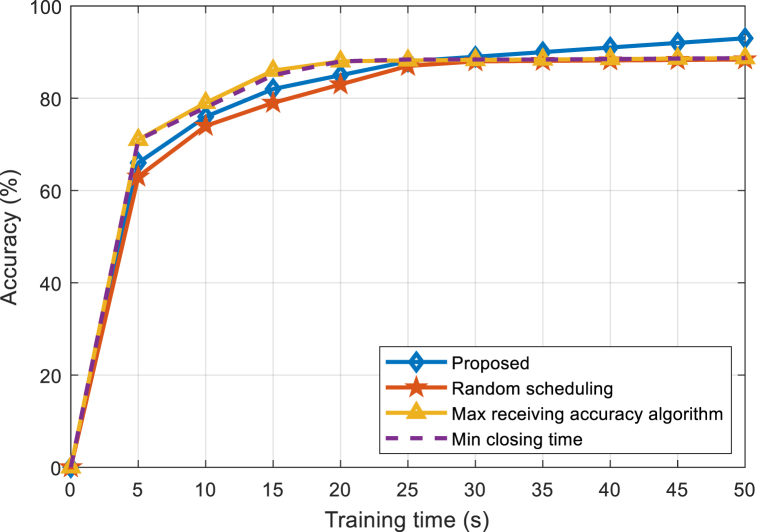
Accuracy comparison between the proposed and existing algorithms under 3 J power.

**Fig. 6:**
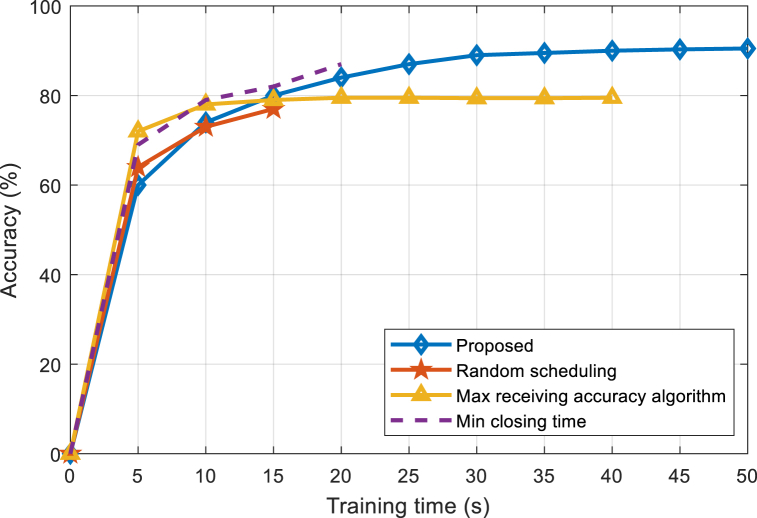
Accuracy comparison between the proposed and existing algorithms under 2 J power.

**Fig. 7:**
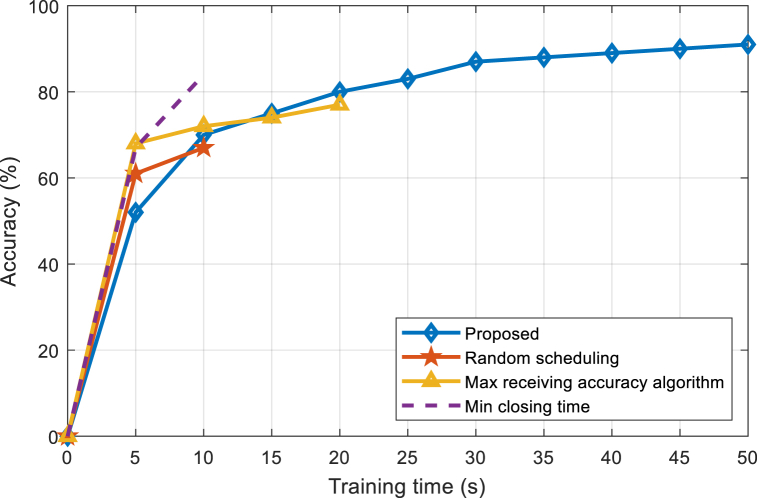
Accuracy comparison between the proposed and existing algorithms under 1 J power.

Fig. 5, Fig. 6, Fig. 7 are the performance comparisons between the proposed algorithm and the benchmark algorithms under 3 J, 2 J, and 1 J battery power respectively. It can be seen that the proposed algorithm has a slow learning speed in the early stage, gradually surpasses other benchmark algorithms in the later stage, and finally converges to the highest accuracy. This is because the set battery power cannot support the device to continuously run at the maximum CPU frequency and maximum transmission power. Among the three benchmark algorithms, the device initially runs at the maximum CPU frequency and maximum transmission power. It is running so the learning speed is high. However, due to the lack of energy management, the battery energy of the device will be exhausted ahead of time and the learning will be interrupted. In contrast, the proposed algorithm learns slowly initially, but thanks to the energy management of the device, the device can train continuously, reaching maximum learning accuracy at the end of the training time. Comparing Fig. 5, Fig. 6, Fig. 7, it can be seen that the lower the battery energy is, the more significant the performance advantage of the proposed algorithm. The lower the battery energy, the earlier the three benchmark algorithms interrupt learning, and the proposed algorithm source managed battery energy has less impact on the final learning accuracy.

**Fig. 8:**
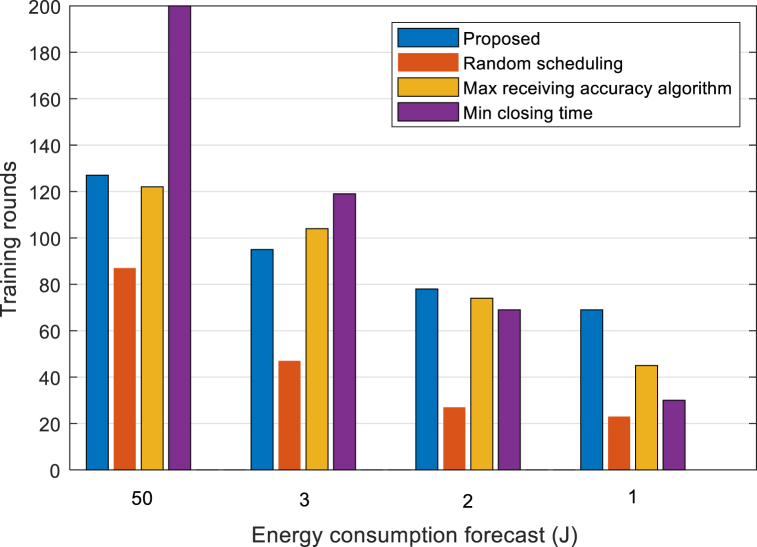
Comparison of training rounds of different algorithms.

**Fig. 9:**
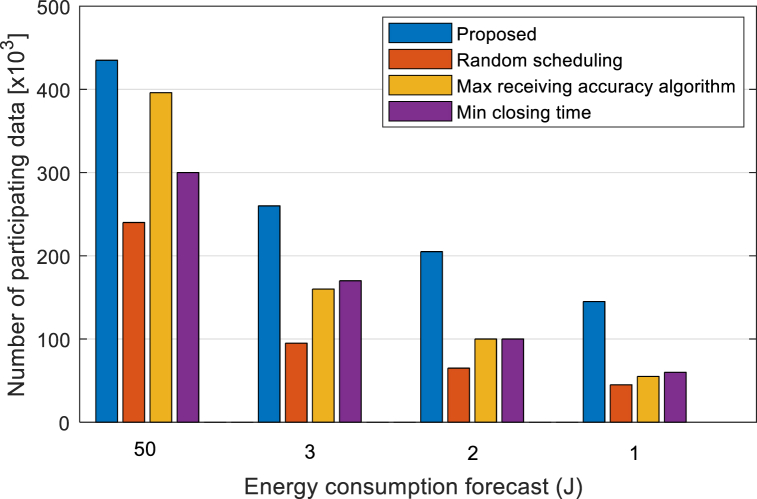
Comparison of the number of data involved in the algorithms under different energy budgets.

To further analyze the performance of the algorithm in this paper under different battery energy budgets, this paper analyzes the training rounds and the number of participating data of different algorithms under a given training time and energy budget, that is, the total number of digital samples involved in the successfully uploaded local model. As shown in Fig. 8, the training rounds of the proposed algorithm increase relatively when the battery energy budget decreases compared with the three benchmark algorithms, so the proposed algorithm can improve the device lifetime. It can be seen from Fig. 9 that at different battery energies, under the budget, the number of sample data successfully participated by the proposed algorithm is three times higher than that of the benchmark algorithms, which reflects the improvement of the training volume of the proposed algorithm. Based on the above evaluation indicators, the simulation results show that the proposed algorithm can well adapt to various battery energy budgets, especially when the battery energy budget is low, and the performance advantage is obvious.

**Fig. 10:**
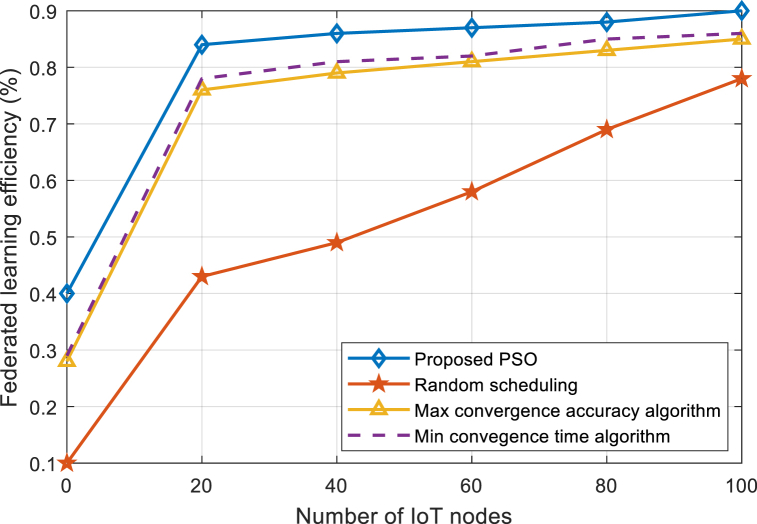
Learning efficiency comparison of PSO and existing algorithms under varying number of IoT nodes.

Fig. 10 compares the learning efficiency of the proposed PSO and existing algorithms with an increasing number of IoT nodes. As can be seen from Fig. 10, the learning efficiency of the algorithms increases with increasing number of IoT nodes. It can also be seen that the proposed PSO algorithm using federated learning has better learning efficiency as compared with existing algorithms.

## 5 Conclusion

Aiming at the problem of limited battery energy and wireless resources in the federated learning network under the IIoT, this paper proposes a resource management algorithm to effectively improve the model accuracy of federated learning under fixed training time. Transform the long-term optimization problem into a dynamic optimization problem, build an online energy allocation strategy, weigh equipment transmission and calculation energy allocation, introduce the concept of learning efficiency, and obtain the learning efficiency under the energy budget resource block matching vectors that maximize efficiency and adjust CPU frequency to save energy. The simulation results show that, compared with the baseline algorithms, the proposed algorithm can improve the learning accuracy of the model, and the learning performance has a significant advantage in the case of energy shortage.

## Author contribution statement

Peizhen Xing: Conceived and designed the experiments; Analyzed and interpreted the data; Wrote the paper.

Hui Zhang: Conceived and designed the experiments; Performed the experiments.

Morched Derbali, Doaa Sami Khafaga: Performed the experiments; Contributed reagents, materials, analysis tools or data.

Shebnam M. Sefat: Conceived and designed the experiments; Performed the experiments; Contributed reagents, materials, analysis tools or data; Wrote the paper.

Amal H. Alharbi, Nor Samsiah Sani: Analyzed and interpreted the data; Contributed reagents, materials, analysis tools or data.

## Data availability statement

Data included in article/supplementary material/referenced in article.

## Funding statement

This research was funded by the 10.13039/501100004515Universiti Kebangsaan Malaysia (Grant code: GUP-2022-060).

This work was sponsored in part by Henan Natural Science Foundation (222102320062).

10.13039/501100004242Princess Nourah bint Abdulrahman University Researchers Supporting Project number (PNURSP2022R120), Princess Nourah bint Abdulrahman University, Riyadh, Saudi Arabia

## Declaration of competing interest

The authors declare that they have no known competing financial interests or personal relationships that could have appeared to influence the work reported in this paper.

## Appendix 1 **:** Proof of Lemma 1

For the theoretical analysis of the federated learning algorithm, this paper adopts the following typical assumptions in the analysis. Assumption 1 : Assume that F(g) satisfies L-smoothness [21,23] which is expressed in Eq. [41]

(41) $$F\left(g_{t+1}\right)\leq F\left(g_{t}\right)+{\left(g_{t+1}-g_{t}\right)}^{T}\nabla F\left(g_{t}\right)+\frac{L}{2}{\left\|g_{t+1}-g_{t}\right\|}^{2}$$

Assumption 2 : Assuming that the degree of dissimilarity between local gradients is bounded under the IID data distribution, the local gradient norm $\left\|\nabla F_{i}\left(g_{t}\right)\right\|$ and the global gradient norm $\left\|\nabla F\left(g_{t}\right)\right\|$ satisfy the B-gradient dissimilarity [13,44], and the local dissimilarity relationship between the gradient and the global gradient is expressed in Eq. [42]:

(42) $${\left\|\nabla F_{i}\left(g_{t}\right)\right\|}^{2}\leq B^{2}{\left\|\nabla F\left(g_{t}\right)\right\|}^{2}$$

According to assumption 1, the expected global loss attenuation satisfies Eq. [43]:

(43) $$E\left(\Delta F\right)\leq E\left(-{\left(g_{t+1}-g_{t}\right)}^{T}\Delta F\left(g_{t}\right)-\frac{L}{2}{\left\|g_{t+1}-g_{t}\right\|}^{2}\right)$$

Further combining Equation [18] and Equation [29], we can get Eq. [44]:

(44) $$E\left(\Delta F\right)\geq E\left(\lambda {\left(\nabla F\left(g_{t}\right)-v_{t}\right)}^{T}\Delta F\left(g_{t}\right)-\frac{L\lambda^{2}}{2}{\left\|\Delta F\left(g_{t}\right)-v_{t}\right\|}^{2}\right)$$

Substituting $\lambda =\frac{1}{L}$ [30–32] into formula [44], we can get Eq. (45):

(45) $$E\left(\Delta F\right)\geq \frac{1}{2L}{\left\|\Delta F\left(g_{t}\right)\right\|}^{2}-\frac{1}{2L}E\left({\left\|v_{t}\right\|}^{2}\right)$$

According to formula [19] and formula [20], the mean value $E\left({\left\|v_{t}\right\|}^{2}\right)$ of the norm square of the difference between the global gradient and the actual global gradient can be obtained as Eq. (46):

(46) $$E\left({\left\|v_{t}\right\|}^{2}\right)=E\left({\left\|\Delta F\left(g_{t}\right)-\frac{\sum_{i=1}^{U}K_{i}a_{i,t}s_{i,t}\nabla F_{i}\left(g_{t}\right)}{\sum_{i=1}^{U}K_{i}a_{i,t}s_{i,t}}\right\|}^{2}\right)$$

Among them, the 0–1 variable $a_{i,t}$ of whether the training device *i* is selected in the *t*-th round, and the 0–1 variable $s_{i,t}$ of whether the transmission is successful is determined by the resource block matching vector $R_{t}^{*}$.

According to [30], $E\left({\left\|v_{t}\right\|}^{2}\right)$ concerning the resource block matching vector $R_{t}^{*}$ and the loss function, $f_{i,k}\left(g_{t}\right)$ can be described as Eq. (47):

(47) $$E\left({\left\|v_{t}\right\|}^{2}\right)\leq E\left({\left(\frac{K_{t}^{no}\sum_{i=1}^{U}K_{i}a_{i,t}s_{i,t}\sum_{k=1}^{K_{i}}\left\|f_{i,k}\left(g_{t}\right)\right\|}{K\sum_{i=1}^{U}K_{i}a_{i,t}s_{i,t}}+\frac{\sum_{i=1}^{U}K_{i}\left(1-a_{i,t}s_{i,t}\right)\sum_{k=1}^{K_{i}}\left\|f_{i,k}\left(g_{t}\right)\right\|}{K}+\right)}^{2}\right)$$

where, $K_{t}^{no}=\sum_{i=1}^{U}K_{i}\left(1-a_{i,t}s_{i,t}\right)$. In this paper, the device data set gradient $\nabla F_{i}\left(g_{t}\right)$ is used to replace the single data gradient $f_{i,k}\left(g_{t}\right)$ combined with the derivation process of formula (47), we can get Eq. (48):

(48) $$E\left({\left\|v_{t}\right\|}^{2}\right)\leq E\left({\left(\frac{K_{t}^{no}\sum_{i=1}^{U}K_{i}a_{i,t}s_{i,t}\nabla F_{i}\left(g_{t}\right)}{K\sum_{i=1}^{U}K_{i}a_{i,t}s_{i,t}}+\frac{\sum_{i=1}^{U}K_{i}\left(1-a_{i,t}s_{i,t}\right)\nabla F_{i}\left(g_{t}\right)}{K}+\right)}^{2}\right)$$

Further, by substituting Hypothesis 2 into Equation (48), we can get Eq. (49):

(49) $$E\left({\left\|v_{t}\right\|}^{2}\right)\leq E\left({\left(\frac{2B\left\|\nabla F\left(g_{t}\right)\right\|\sum_{i=1}^{U}K_{i}\left(1-a_{i,t}s_{i,t}\right)}{K}\right)}^{2}\right)$$

Based on the Cauchy-Schwarz inequality [21], satisfying ${\left(\sum_{i=1}^{U}K_{i}\left(1-a_{i,t}s_{i,t}\right)\right)}^{2}\leq U\sum_{i=1}^{U}{\left(K_{i}\;\left(1-a_{i,t}s_{i,t}\right)\right)}^{2}$, substituting into formula (49), Eq. (50) can be obtained:

(50) $$E\left({\left\|v_{t}\right\|}^{2}\right)\leq E\left(\frac{4B^{2}{\left\|\nabla F\left(g_{t}\right)\right\|}^{2}U\sum_{i=1}^{U}{\left(K_{i}\;\left(1-a_{i,t}s_{i,t}\right)\right)}^{2}}{K^{2}}\right)$$

Since $a_{i,t}$ is a 0–1 variable, there are $a_{i,t}^{2}=a_{i,t},E\left(s_{i,t}\right)=q_{i,t}$, further can be obtained as Eq. (51):

(51) $$E\left({\left\|v_{t}\right\|}^{2}\right)\leq \frac{4B^{2}{\left\|\nabla F\left(g_{t}\right)\right\|}^{2}U\sum_{i=1}^{U}K_{i}^{2}\left(1-a_{i,t}\left({2q}_{i,t}-q_{i,t}^{2}\right)\right)}{K^{2}}$$

Simultaneously according to (45), (51), the lower limit of expected global loss attenuation can be obtained as Eq. (52):

$$E\left(\Delta F\right)\geq \frac{1}{2L}{\left\|\nabla F\left(g_{t}\right)\right\|}^{2}-\frac{1}{2L}E\left({\left\|v_{t}\right\|}^{2}\right)$$

(52) $$\geq \frac{1}{2L}{\left\|\nabla F\left(g_{t}\right)\right\|}^{2}\left(1-\frac{4UB^{2}}{K^{2}}\sum_{i=1}^{U}K_{i}^{2}\left(1-a_{i,t}\left({2q}_{i,t}-q_{i,t}^{2}\right)\right)\right)$$

## Appendix 2 **:** Proof of Lemma 2

Based on the delay definition formula [7], it can be known that the delay monotonically decreases as the device transmit power or CPU frequency increases.

To prove Lemma 2, it is only necessary to prove that the definition of energy consumption [9] is a monotonically increasing function of the transmit power and CPU frequency which is expressed as Eq. (53):

$$e_{i,t}=\delta_{i}{\bar{\omega }}_{i}f_{i,t}^{2}\varepsilon_{i}K_{i}+\frac{P_{i,t}}{\sum_{n=1}^{M}r_{i,n,t}B^{Up}\;\log\left(1+\frac{P_{i,t}h_{i}}{I_{n}+B^{Up}N_{0}}\right)}$$

(53) $$=\delta_{i}{\bar{\omega }}_{i}f_{i,t}^{2}\varepsilon_{i}K_{i}+\frac{\ln\;2\left(I_{n}+B^{Up}N_{0}\right)}{B^{Up}h_{i}}\frac{x}{\sum_{n=1}^{M}r_{i,n,t}\;\ln\left(1+x\right)}$$

where, $x=\frac{P_{i,t}h_{i}}{I_{n}+B^{Up}N_{0}}$.

Since the calculation energy consumption is proportional to the square of the CPU frequency, the definition of energy consumption (formula [9]) is a monotonically increasing function of the CPU frequency. The first derivative of energy consumption concerning the transmit power is expressed in Eq. (54):

(54) $$\frac{\partial e_{i,t}}{\partial P_{i,t}}=\frac{\ln\;2}{B^{Up}}\frac{\sum_{n=1}^{M}r_{i,n,t}\left(\ln\left(1+x\right)-\frac{x}{1+x}\right)}{{\left(\sum_{n=1}^{M}r_{i,n,t}\ln\left(1+x\right)\right)}^{2}}$$

Among them, let $f\left(x\right)=\ln\left(1+x\right)-\frac{x}{1+x}$ .

Since x>0 when $f^{\prime }\left(x\right)=\frac{x}{{\left(1+x\right)}^{2}}>0$, and f(0)=0, f(x) is always positive when x>0, further, the first-order energy consumption with respect to the transmission power derivative is always positive when the transmitted power, $P_{i,j}>0$, that is, the energy consumption increases monotonously with respect to the transmitted power $P_{i,t}$.
